# Characteristics and impacts of live music interventions on health and wellbeing for children, families, and health care professionals in paediatric hospitals: a scoping review

**DOI:** 10.1080/17482631.2023.2180859

**Published:** 2023-03-07

**Authors:** Anna-Karin Kuuse, Ann-Sofie Paulander, Louise Eulau

**Affiliations:** a Department of Nursing Science, Sophiahemmet University, Stockholm, Sweden; b Royal College of Music in Stockholm, Department of Music Pedagogy, Stockholm, Sweden

**Keywords:** Families, health care professionals, live music intervention, paediatric hospital, review, well-being

## Abstract

**Purpose:**

The objective of this scoping review is to compile and examine characteristics and impacts of live music interventions on the health and wellbeing of children, families, and health care professionals in paediatric hospital care.

**Methods:**

We searched four scientific databases for peer-reviewed publications of empirical studies of all study designs. The first author screened the publications, with spot-checks for eligibility by the second and third authors. Data extraction and quality assessment were made by the first author with support from the second and third. Additionally, the included studies were screened for quality appraisal. The analysis followed an inductive, interpretive approach for synthesis.

**Results:**

Quantitative features were screened and compiled, and qualitative inductive analyses of findings were elaborated into categories connected to research questions. The reported impacts were thematized through emergent features of importance and prerequisites beneficial for successful interventions. Recurrent outcomes present themes of *positive affect, coping*and *reduced hospitalization*. *Emotional regulation, play and participation, age, session design, adaptivity, and familiarity* present benefits, barriers, and facilitators for outcomes.

**Conclusions:**

Findings from collected empirical research display philosophy, practice, and relations as keys for characteristics, impacts, and implications of live music interventions in paediatric hospital care. The communicative aspects of music appear at the core of importance.

## Introduction

Due to an increasing awareness of the beneficial impacts from art-based activities on health and wellbeing, this scoping review deals with live music interventions for children and their families in the context of paediatric hospital care. Concerning musical activities, music therapy and music performed by professional musicians seem to be taking the lead in the field (Dileo & Bradt, 2005; Fancourt & Finn, 2019, Robb & Carpenter, 2009). Music-based activities may include active music engagement (e.g., playing and singing) and listening to both live and pre-recorded music (Dileo & Bradt, 2005). As a non-pharmacological health care intervention, music-based activities are often considered cost-effective and, especially for children, motivated by a lesser need for medication (e.g., sedatives and analgesics) (Johnson et al., 2021). Other common targeting areas for music delivery in paediatric health care are: distraction from the hospital environment (Vernisie, 2015), management of distress and pain (Holochwost et al., 2020; Pope, Tallon, McConigley, & Wilson, 2015), emotional regulation (Archambault et al., 2019; Tucquet & Leung, 2014), as well as overall wellbeing (Cheung et al., 2021; O’callaghan et al., 2013), not least from a child- and family-centred care perspective (Davidson et al., 2017; Lindenfelser et al., 2012; Ullsten, 2019). Altogether, there is a growing body of evidence indicating beneficial effects of music interventions for children in paediatric health care, but more rigorous research is still needed to examine prerequisites for both practical and professional implications (Stegemann et al., 2019). The lack of more generalized definitions and standardizations of musical interventions for health and well-being in the paediatric population further limits conclusions on their effectiveness (Naylor et al., 2011; Robb & Carpenter, 2009, 2019).

Among the musical interventions reported in paediatric health care, music therapy is still the profession most often referred to (Robb & Carpenter, 2009). However, interventions are occasionally delivered by other health professionals. To date, there is also more significant evidence for positive impacts from interventions led by a board-certified music therapist (Treurnicht Naylor et al., 2011), partly explained by extensive professional training regarding a person-centred design (Stegemann et al., 2019). However, during the past 10–15 years, a new music profession has been slowly emerging within paediatric hospital care, i.e., *the health musician* (Koivisto & Tähti, 2020; Ruud, 2013), targeting more general claims of health and wellbeing with the help of live music interventions. Following Ruud (2013), the establishment of this profession may be perceived as an effect of a general cultural/sociological shift in the field of music and health towards a more community-based, health-promoting approach. This philosophical turn raises new implications for future objectives coupled to live music interventions for health and wellbeing. However, the displayed heterogeneity of music therapy interventions (Robb & Carpenter, 2009) and the implementation of a new music profession—not yet professionally and scholarly evaluated (Koivisto & Tähti, 2020) – still seem to re-actualize the call for a conceptual framework (Shoemark et al., 2018) and thorough elaborations of health-related outcomes connected to live music interventions (Stegemann et al., 2019)

## Health and wellbeing

According to recently updated policies, the World Health Organization defines health “as a state of complete physical, mental, and social well-being and not merely the absence of disease or infirmity” (Fancourt & Finn, 2019). This clearly emphasizes the interdependence between all the domains of wellbeing. The notion of a fixed or “complete state” has recently been elaborated in favour of a more salutogenic perception of health, also counting on-going experiences of coherence and meaning as prerequisites (Antonovsky & Sagy, 2017). To perceive health and wellbeing as mere salutogenetic processes does not exclude measurable biological features, such as quantitative signs of impacts on health. Still, the new definitions highlight the need for a more ecological and process-oriented approach to interventions aimed at health and wellbeing (Ansdell & DeNora, 2012). When defining the concept of health and wellbeing for this review, a salutogenetic interpretation is at the fore and studies included have addressed these considerations. This implies that what matters in relation to health and wellbeing “is not only how poorly we feel, but it is of no less importance how well we feel, and which cultural resources are available to sustain and develop our sense of coherence” (R. MacDonald et al., 2013, p. 6.).

Concerning wellbeing, children are often examined broadly as a group (Cho & Yu, 2020). However, the growth and psychological development that a person undergoes at the beginning of their lifespan demands further subdivisions. In ’Infancy’, i.e., the period between birth and age 2, it is primarily the family that provides the nurturing care, while school and the surrounding society gradually become more important during ’Childhood’ (ages 1–2 to 12–13^1^) (Yousafzai, 2018). Adolescence, on the other hand, is a transitional phase of rapid physical, cognitive, and psychosocial growth and development that takes place between ages 10 and 19.^2^ The World Health Organisation (WHO) defines ’Adolescents’ as: “individuals in the 10–19 year age group and ‘Youth’ as the 15–24-year age group, while ‘Young People’ covers the age range 10–24 years”.^3^ In an attempt to describe well-being of the adolescent from the perspective of childhood, Gennings et al. (2021) define it as: “a multifaceted perception of an interaction between an individual’s positive feelings and external influences” (p.84). This is in line with a concept analysis concerning wellbeing in childhood that includes overall positive emotions, positive self‐esteem, and resilience (Courtwright et al., 2020). Family and social connectedness have an important place in the literature of wellbeing at the beginning of the lifespan. This also points to the need to consider a child- and family-centred perspective on wellbeing in paediatric hospital care, especially for very young children.

## Live music intervention and health

In this review, the medium of music is understood to be coupled to social, cultural, and psychological variables, and that musical experiences go beyond the perception of sounds, while also influencing affects, thoughts, and behaviours (Elliott & Silverman, 2013). One important foundation of this perception lies in the notion of music as a communicative feature, psycho-biologically inherited, although socially developed (Malloch et al., 2012). This gives the interactive processes in live music an important intersubjective and cultural meaning through action and performance (Stige, 2013). When coupled to the concepts of health and wellbeing outlined above, live music interventions are thus perceived as intersubjective health resources supporting the development of coping strategies regardless of conditions, and as potential opportunities for the experience of social, cultural, and psychological coherence (R. MacDonald et al., 2013). Despite the diversity of frames for the interpretation of outcomes, such claims still imply that the potential of live music intervention as a health resource lies in the interdisciplinary understanding of music, health, and wellbeing as ecological phenomena (Ansdell & DeNora, 2012).

Following the outlines of the conceptualizations of music, health, and wellbeing, this study sets out to trace common features of live music interventions in paediatric hospital care regardless of profession, design, and objectives. As far as we are aware, there is no previous review of empirical research studying common outcomes of health and wellbeing connected to live music interventions, delivered by several professionals, in paediatric in-patient hospital care settings. The emerging scholarly field of music, health, and wellbeing, which is evolving philosophically, may subsequently create spaces for new professional claims and objectives. By drawing conclusions and comparing objectives, design, protocols and reported outcomes from several kinds of live music interventions in paediatric hospital care from the past decade, we wish to contribute to an ongoing negotiation and re-conceptualization of music for health and wellbeing. This scoping review sets out to examine common findings across populations with the aim of charting how live music interventions, delivered by music therapists, musicians, or other professionals, are perceived as beneficial for children and young patients, families, and on-site health care professionals. Additionally, we have included studies with perspectives from participating musicians. Contemporary conceptualizations of music, health, and well-being point to an approach driven by theory (FHM Folkhälsomyndigheten/Swedish Public Health Authority, 2017).

## Objective and questions

The objective of this scoping review is to compile and examine characteristics and impacts of live music interventions on health and wellbeing for children, families, and health care professionals in paediatric hospital care. The ambition is to map various perspectives and to draw some interpretive conclusions concerning trends in the execution of live music for paediatric health and wellbeing in empirical research. The overarching question is: In what ways are live music interventions and their outcomes described, assessed, and perceived to act as resources for health and wellbeing for children, families, and health care professionals in paediatric hospital care? The following sub-questions will be addressed operationally:

1. Which populations are included in empirical research on live music interventions in paediatric hospital care?
2. What kinds of design and methodology are used in these studies?
3. What kinds of musical activities are studied?
4. Which profession delivers the live music intervention?
5. What kinds of impacts related to health and wellbeing are discussed?
6. What perspectives from families, music intervenors, and paediatric health care professionals are presented?
7. Which aspects of live music interventions in paediatric hospital care appear as beneficial and potentially promising for health and wellbeing for the intended populations?
8. Which barriers and facilitators are illuminated?
9. How sound is the scientific quality of included studies?

## Materials and methods

Scoping reviews are especially useful when examining the magnitude and character of literature in a certain area of research. They are also useful in addressing more exploratory research questions (M. Peters et al., 2015). When compiling, summarizing, and communicating research results for practice, such a review often illuminates gaps in knowledge concerning a specific phenomenon. According to the Swedish Public Health Authority (FHM Folkhälsomyndigheten/Swedish Public Health Authority, 2017), a scoping review can thus be used to assess barriers and facilitators within different societal implementations, as well as to show the need for further studies, or a more comprehensive systematic review. Research collected in this article is heterogeneous with a broad range of findings, which supports an initial examination of the field with the help of a scoping review. As included studies use both quantitative and qualitative designs, together with a descriptive ambition, a more interpretive stance influenced by theory has been exhibited while presenting results. The present study follows the methodological framework for scoping reviews recommended by the Joanna Briggs Institute (M. D. Peters et al., 2015) and (2017).

## Eligibility criteria

### Participants and perspectives

We have included all participants within the field of live music interventions in paediatric hospital care. While not discriminating between subgroups surrounding children and young people receiving the interventions, we widened the scope of the study to the literature on child/young patients, families to child/young patients, health care professionals, and musicians/music therapists working in paediatric care. We combined two different but complementary frames for a scoping review. The Joanna Briggs Institute (JBI) framework suggests PCC (*Participant, Concept and Context*) for the development of eligibility criteria, whereas the Swedish Public Health Authority (FHM) framework suggests SPICE (*Setting, Perspective, Intervention, Comparison, Evaluation*) as a template for the elaboration of the entire study. Additionally, the SPICE model recommends a theoretical framework to facilitate identification of important aspects of research questions (FHM Folkhälsomyndigheten/Swedish Public Health Authority, 2017). Combined, the heading *Participant* thus also indicates whose *Perspective* was taken. In this review, children and young patients in paediatric hospital care (age 0–17) are included as the core population to consider, while focusing on health and wellbeing with the help of musical activities. We excluded studies on children in neonatal intensive care units (NICU), and studies targeting patients older than 17 years. Families, health care professionals, and musicians/music therapists are included subgroups whose perspectives are considered highly important for the characteristics and outcomes along with the live music interventions performed.

### Concepts and interventions

*Live music intervention, health* and *wellbeing* were core concepts examined. We searched especially for empirical research examining effects on aspects related to perceived outcomes, and assessable features of health and wellbeing connected to live music interventions in children’s hospitals. A broad range of assessments emerged: biological measures (heart rate, saliva, hormones), behavioural measures (distress, pain perception, mood, facial expressions) as well as other qualitative and quantitative, observatory and interpretive outcomes related to health and wellbeing. As live music intervention, health and wellbeing are concepts considered to be under pending philosophical negotiation (R. A. MacDonald, 2013) we still aimed to find studies where they were sufficiently outlined. For a study to be included, we wanted the live music activities to be the core focus, interventions which, in form and action, could be delivered by a musician, music therapist or other professional. We still included studies where different kinds of live music interventions were compared with receptive music interventions (e.g., music listening). Additionally, studies where a live music intervention was compared with other live interventions were included (e.g., clowns, dogs, artwork and reading). Studies in which it could not be determined whether the core music intervention was live or recorded, whether the music intervention was solely about listening to pre-recorded music, and whether the study was a review or a theoretical elaboration on the issues for examination, were excluded.

### Context and setting

There are several out-patient paediatric settings where children and young people can meet health care professionals. This scoping review includes studies of live music interventions in paediatric hospital settings, which were performed with the intention of affecting aspects of health and wellbeing. We excluded studies not performed in paediatric hospital care. However, while focusing on the very broad concepts of health and wellbeing, we still included studies from a wide range of in-patient paediatric hospital care for children and young people (age 0–17), although kept to contexts and settings where different features connected to music, health and wellbeing have been sufficiently outlined (e.g., distress, anxiety, pain perception, emotional regulation, agency and participation).

## Information sources and search strategy

The literature search, made in October and November 2021, with a complementary update in March 2022, was conducted by the first author with support from the author group and supervision from two research librarians. Initially, we searched the field for previous reviews in English with similar scope. An initial pilot search for peer-reviewed reviews in the databases Prospero, Cochrane Library and PubMed from year 2000, and with the keywords “Music AND Children AND Hospital” gave 158 hits, where 32 studies and protocols first appeared to be eligible. Most stemmed from 2010 and onwards. With the help of the two librarians, a more comprehensive search strategy was applied including participants, concepts, and context (Table I). In these searches, we further expanded the timeframe for PubMed to 1990–2021 but still found no eligible reviews published between 1990 and 2011. After duplicate control, we found 59 review studies between 2011 and 2021, of which 27 met eligibility criteria. Additionally, we searched Google Scholar and ERIC for review studies that may have been missed. Four new potentially eligible studies were found. The first author screened the 31 reviews for the full text, but none met all the criteria. However, one systematic review (Treurnicht Naylor et al., 2011) adopted a broader stance, looking at the effectiveness of music interventions in paediatric healthcare, although included only randomized control trials. Similarly, it was not clearly outlined whether the interventions assessed were live or receptive. Despite the lack of an apparent match, the scope of the review was close enough to constitute a stepping-stone. At that time, we initiated the screening for empirical research meeting the eligibility criteria. This was made in two rows. Firstly, we searched for peer-reviewed studies in English within the four databases, PubMed, Academic Search Elite, Psycinfo and Cochrane Library between 2011 and 2021. Search strings were modified to fit with each database. In the second row, we made a complementary search in PubMed covering 1990–2010. After this process, one of the potentially eligible research studies (Preti & Welch, 2011) also appeared to have approximately the same scope as ours. With this, a final argument for timely framing emerged. In the light of a lack of previous reviews with a similar scope, Treurnicht Naylor et al. (2011) still showed some eligible objectives. The latter, together with the empirical study of Preti and Welch setting out to present the emerging field of professional musicians in health care services (Preti & Welch, 2011) decided us to limit screening to 10 years (2011–2021), also a suitable timeframe when screening for contemporary research evidence in PubMed and other databases.

**Table I.** Search strategy for PubMed database.

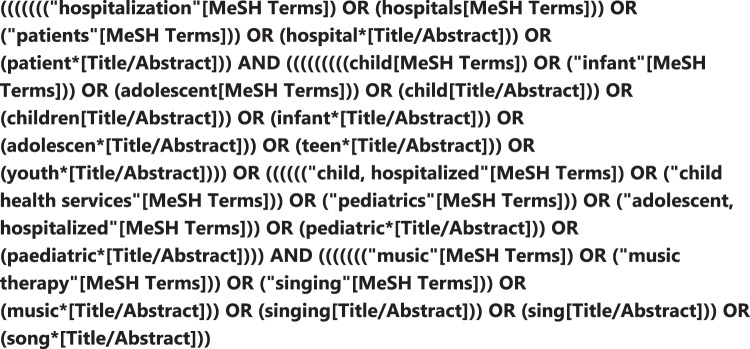

## Selection of sources of evidence

**Figure 1.**
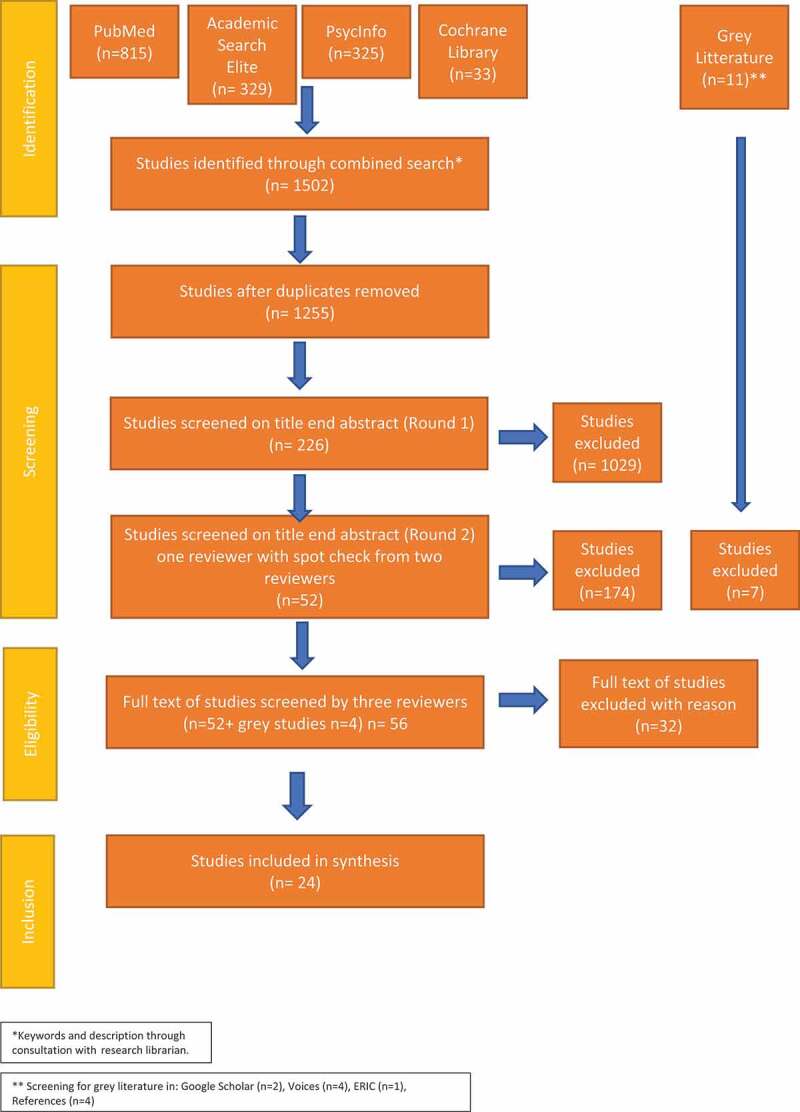
PRISMA flowchart of study selection.

Citations were transferred to the web-based citation software Endnote. In the first round, the first author screened abstracts and titles for eligibility, i.e., following the inclusion/exclusion criteria. In the second round, members of the research group made spot-checks for eligibility. Fifty-six studies met the eligibility criteria and were screened for full text, wherein four grey studies were included (FHM Folkhälsomyndigheten/Swedish Public Health Authority, 2017). Concerning trustworthiness (Elo et al., 2014), all three authors were involved in every phase of the analysis process, including the preparation, organization, and reporting of results. For instance, in the process of selecting sources, the second and third authors made spot-checks, screening five studies each. The complementary search in March 2022 yielded one additional study. Finally, 24 studies were included for review. The entire process of identification, screening and eligibility checks for empirical studies is illustrated in a PRISMA flow chart (Figure 1). Those reviews and studies excluded were stored as relevant for future use. Following Robb et al. (2011) and Stegemann et al. (2019), design and findings in previous empirical music intervention studies are reported to be extensively heterogeneous with a high proportion of methodological flaws. To support critical reflections of findings, we decided to include the execution of quality appraisal despite this not being required in scoping reviews.

## Data charting process and quality appraisal

To chart and evaluate a variety of populations, countries of origin, designs, methods, objectives, and findings in the included studies, a matrix of information was produced for an overview (Table II). Following research questions 1–5, different countable features described in the included studies were compiled. Here the objective was to chart the prevalence of features, searching for visible trends. Regarding research question 9, each study was screened for quality appraisal with the help of a MetaQuat tool from Public Health Ontario (Rosella, 2015), one of a few recommended templates when compiling results from quantitative, qualitative, and mixed method research (FHM Folkhälsomyndigheten/Swedish Public Health Authority, 2017). When screening each study for quality in the categories: relevancy, reliability, validity and applicability, a final assessment was made showing high/medium/low/extremely low quality (Table III). Here, we considered previous critical examinations of music intervention studies while additionally giving space to reflections in relation to theory. The next round of analysis was qualitative. A content analysis of findings (Patton, 2015) was made both in relation to research questions (question 5–8) inductively searching for unforeseen themes and categories among reported experiences and impacts. Following the methodology of qualitative content analysis (ibid.), all studies were first screened for the collecting of codes in relation to the operational questions (impacts, subgroups perspectives, promising aspects, barriers, and facilitators). Some codes falling outside of the research questions were coded as interesting for potential meta-categories. All codes were marked and subsequently synthesized into themes, thus also displaying how often they occurred. Finally, themes were categorized, again in relation to the structure of research questions.

**Table II.** Overview of studies included.

Authors/ year/country/reference	Participant characteristics (n, age)	Study design/ Data collection method	Music intervention Activities/profession in charge	Intervention objectives, hypothesis, issues under consideration	Findings/Reported benefits/ effectiveness, Promising components
1)Antonelli et al. (2019)./Italy. **A Comparison of Non pharmacologic Interventions on the Emotional State of Children in Emergency Department**. *Paediatric Emergency care.*	Children aged 3–16 years (*n* = 108) Boys (*n* = 54), Girls *n* = 51): Experimental group (*n* = 57) with: Clowns (*n* = 18), dogs (*n* = 24), musicians (*n* = 15) + control group (*n* = 48)	Quantitative design: Positive and negative affect schedule (PANAS) = Self-reported measures from parents about kids. PANAS -C for children. Self—assessment manikin (SAM), pictorial assessment for to evaluate children’s´ affective reactions Wong-baker scale (Faces Scale) = child self-reported pain Parents´ Evaluation Questionnaire Health professionals´ Questionnaire	Live music interaction with one musician for 15 children and their parents	Investigate the efficacy of 3 different non-pharmacologic interventions (clowns, dogs, and musicians) to reduce pain and analyse the perception of positive and negative affects after the presence of these activities. To measure the emotional state of children in the emergency department after the interaction and their impact on pain perception/evaluate the emotional state of parents/investigate the parents´ and the health professionals´ opinions and knowledge on the presence of the interventions.	All interventions seem to empower positive affects in children but not influence the self-reported pain. Differences among groups did not emerge—confirms the equivalent quality of the 3 different activities. Parents rated the musicians lower; health professional sees music as interfering with care.
2)Blackburn, 2020a/UK **My Hospital Family: Family perceptions of a Singing Medicine project in a Children´s Hospital in England**. *Nursing Children and Young People*	Children ex-patient (*n* = 1), Children caregiver (sister) (*n* = 1), Adult caregiver (*n* = 4)	Qualitative (semi-structured interviews) Ecological Systems and Froebelian Theory	Singing games with Vocal tutors	Family perceptions of their child´s participation in participatory singing games for children with a range of health care needs at children´s hospital: What are the views and perceptions of family members on the role of musical activities and games during their child´s stay in Hospital? How can the application of ecological systems theory and Froebelian principles help in understanding the meaning of these findings?	Characteristics of Vocal tutors (funny, gentle, emotive etc.) Singing games affecting emotions and mental wellbeing Hospital family- a normalizing experience Distraction—happy memories Lasting effects on family relations
3) Blackburn (2020b)/UK **“The people in purple shirts´”: Froebelian insights to a Singing medicine project in a children´s hospital**. *Journal of Early Childhood Research.*	Caring staff of children patients (*n* = 13)	Qualitative/interpretative study, Observations, semi-structured individual interviews	Individualized Singing Medicine delivered by singing tutors at wards and bedside—experiences and observations from staff	To explore the role of a SM project in a children’s hospital and to understand how the application of Froebelian principles can help us understand and conceptualize children´s rights and well-being in restricted environments such as Children´s Hospital. What are the views and perceptions of health and education professionals who work in the Hospital on the role of music and singing games in children´s experience and recovery of short and long-term health conditions? How can participatory arts projects in a children´s hospital bring new understanding to Froebelian principles?	Participants value the human connectedness imbued by the Singing Medicine project. Participating in the SM singing games has been demonstrated to benefit children´s well-being, right to make choices and engage in playful activities. The findings can be mapped against all six of the Froebelian principles.
4) Bush et al. (2021)/US. **Effect of Live versus Recorded music on Children receiving mechanical Ventilation and Sedation**. *Paediatric Critical Care*	Children patients aged 0–2 years (*n* = 33)	Explorative randomized controlled trial/quantitative design with measures of heart rate, respiratory rate, mean arterial pressure (MAP) before (2 times) and after (5 times) interventions—live music group (*n* = 17) or recorded music group (*n* = 16)	Live music intervention by MT guitar and voice—matching/adapting to the child. Recorded music intervention by MT with same songs	To determine the effect of a live music intervention versus a recorded music intervention on heart rate, blood pressure and respiratory rate in paediatric critical care patients receiving mechanical ventilation and sedation	Live music interventions may be more effective than recorded music intervention in reducing pain and anxiety in paediatric critical care patients. The advantage of live music may be due to the adaptability of the music delivery by a trained music therapist. Data shows a significant decrease in heart rate in the live music intervention group compared with the baseline measurement.
5) Colwell et al. (2013)/US. ***Impact of Music Therapy Interventions (Listening, Composition, Orff-based) on the Physiological and Psychosocial behaviours of Hospitalized Children: A Feasibility Study**. Journal of Pediatric Nursing*	Children patients aged 6–17 (*n* = 32, 17 females, 15 males)	Qualitative design/Feasibility study. Pre-and post-measures of heart rate, blood pressure, oxygen saturation Wong-Baker Faces pain rating scale. The State-trait anxiety inventory for children (STAIC) Video-analysis of behaviours/level of engagement: eye contact (with therapist or materials), facial affect (positive, neutral, negative), verbal interactions (responding to questions/choices, initiating conversation), participation (on-task, verbal off-task, motor off-task, passive off-task) and length of session task (duration)	Overall theme: all about me—focus on children as individuals, allow opportunities for emotional expression, social interaction, choices, and decision making, and verbal processing of creative choices Music listening group—Choose and listen to preferred music from a list (45 min) Music composition group—compose on computer and create a CD label (45 min) Music playing session—Orff-process for playing and improvising (unpitched and melodic percussion instruments, speech chanting, body percussion)	To compare three music therapy strategies (listening, composing, and Orff-based) on physiological and psychosocial behaviours of hospitalized children. Outcomes can indicate effectiveness of various coping strategies	No clinical significance was indicated as a result of treatment on heart rate, blood pressure, or oxygen saturation but there was a decrease in self-reported pain and anxiety. There was not much differentiation among interventions. Videotape analysis determine level of engagement in coping-related behaviours
6) Giordano et al. (2021)/Italy **Covid-19 and absence of music therapy: Impact of mother-child dyad during invasive procedures in paediatric oncology**. *The Arts in Psychotherapy*	Children patients aged 2–15 (*n* = 20) in dyads with parent	Quantitative study/questionnaire with possibility for open-ended question	Bedside Music therapy intervention from certified MT (relational, free improvisational, receptive) before IP and until analgo-sedation.	To compare the level of anxiety and sedation in paediatric patients undergoing IP with and without the presence of MT before, during and after COVID-19 pandemic. Secondary aim was to assess mothers´ anxiety level	The study demonstrates a clear contrast between pre-procedural anxiety when the music therapist was present, and when he was absent due to the COVID-19 outbreak. These effects were observed both in the patients and their mothers, with MT having a positive impact on the mother-child dyad. The combination of reduced anxiety and sedation in children who received MT compared to those who did not, suggests that MT can be an effective, complementary intervention for paediatric patients undergoing invasive procedures.
7) Giordano et al. (2020)./Italy **The influence of music therapy on preoperative anxiety in paediatric oncology patients undergoing invasive procedures**. *The Arts in Psychotherapy.*	Children cancer patients aged 2–13 (*n* = 48) in dyad with parents	Quantitative study/control study: The modified Yale Pre-operative Anxiety Scale (m-YPAS) (Observational behavioural checklist) Questionnaire to Staff	Music Therapy intervention (*n* = 29) (1–6 sessions at bedside, interactional relational approach, active and receptive techniques) Standard care (*n* = 19) (entertainment with leisure activities)	To evaluate the influence of MT as a complementary/non-pharmacological intervention to reduce preoperative anxiety and promote more compliant behaviours during anaesthesia induction.	A lower preoperative anxiety score in the MT group compared with standard care was observed. Results support the potential effectiveness of integrating music therapy with a pharmacological approach to reduce preoperative anxiety in IP. More than 90% of the medical staff (*n* = 19) were satisfied with the ability of MT to distract the patient and support the staff.
8) Grebosz-Haring and Thun-Hohenstein (2018)./Austria. **Effects of group singing versus group music listening on hospitalized children and adolescents with mental disorders: A pilot study**. *Heliyon.*	Children patients aged 11–18 (*n* = 17, 11 females, 7 males)	Quantitative observational pilot study Baseline: Psychiatric condition, medication, therapies + patient questionnaire sociodemographic data and musical background Multidimensional Mood questionnaire (MDBF) before and after music intervention Warwick-Edinburgh mental wellbeing scale (WEMWBS) Paediatric Quality of Life Inventory 4.0 Generic Core Scale Child Self Report Version (PedsQL) Biological outcomes: salivary cortisol and salivary IgA Patient self-report question- subjective perception of Music intervention	Singing lessons with professional choirmaster with therapeutic background—breathing, relaxation, songs of various style (45 min). Music listening group session provided by a trained music therapist without therapeutic relationship—muscle relaxation, calm instrumental music (45 min).	Hypothesis: Compared with music listening condition the short time five-day programme of group singing would lead to more positive changes in psychological outcomes such as improvement of self-reported mood state, patient wellbeing and quality of life and would induce positive changes in hormone and immune responses, such as an increase in salivary IgA levels (immune response) and a reduction of salivary cortisol (stress response)	Singing group intervention—larger significant decrease in cortisol levels = Active group singing might have calming and stress-reducing effects Listening group intervention- larger significant increase in psychological measures of mood state, in the dimension calmness Several limitations and confounders are discussed.
9) Longhi et al. (2015)/Italy/UK **Wellbeing and hospitalized children: Can music help?** Psychology *of Music.*	Children patients (*n* = 37)	Quantitative design (Oxygen saturation level, heart rate, pain assessment)	Every child participated in 3 × 10-minutes sessions of each: 1) Music, 2) Reading, 3) No interaction	To understand the effects of music on preschool children with cardiac and/or respiratory problems. In particular, to investigate the social component of the music sessions: to ascertain whether it is the music per se, or the adult attention associated with it that might improve their state.	Physiological responses and pain assessment at the end of the music session suggest that the patients felt lower levels of anxiety and pain as compared to other (non-music) sessions—live music can help to distract, relax, and reduce anxiety for children as well as reduce pain. It seems to be live music per se, and not the social component of the musical interaction …
10) Millett, 2017,2017/US. **Comparing Active and Passive Distraction-Based Music Therapy Interventions on Preoperative Anxiety in Pediatric Patients and Their Caregivers**. *Journal of Music Therapy.*	Paediatric patient and caregiver dyads (*n* = 40)	Quantitative design: The modified Yale Pediatric Anxiety Scale (mYPAS) for children Strait-Trait Anxiety Inventory-Y6(STAI-Y6) for caregivers Randomized groups for active (*n* = 19) or passive music therapy (*n* = 21)	Active intervention: 15 minutes patient- and family-preferred music or age-appropriate music with music therapist (guitar, percussion-instruments, songs and games) Passive intervention: 15 minutes music-assisted relaxation, patient- and family-preferred music.	Is MAE (active intervention) or MAR (receptive intervention) more effective in reducing preoperative anxiety in paediatric patients and their caregivers? Are preoperative music therapy interventions, regardless to specific intervention, effective in reducing preoperative anxiety in paediatric patients and their caregivers?	Both active and passive music therapy interventions may be equally effective in reducing anxiety prior to surgical operations. Researchers´ suggestion is that interventions be matched to the child´s temperament, presentation, and family values to determine whether an active or passive approach would be most beneficial.
11) Ortiz et al., 2017/US. **Impact of a Child Life and Music Therapy Procedural Support Intervention on Parental Perception of Their Child´s Distress During Intravenous Placement**. *Paediatric Emergency Care.*	Parents of Children patients aged 4–11 (*n* = 41)	Quantitative (mixed method) design, Likert—type scale with 4-question pre-test and 4-question post-test Qualitative data with open ended comments	Individualized live music intervention by MT as receptive music during preparation, Live music therapy intervention during placement, live music therapy intervention as processing after placement	To measure the efficacy of a multimodal approach involving child life and music therapy interventions that address procedure-related distress in paediatric patients by parental perception. Uncomfortable/Afraid/Cooperative/Calm	Improvement was demonstrated across all 4 questions suggesting that the child life and music therapy intervention supported healthy, adaptive coping and helped to minimize distress experienced by patients during IV placement
12) C. Preti and G. F. Welch (2013)/Italy. **Professional identities and motivations of musicians playing in healthcare settings: Cross-cultural evidence from UK and Italy**. *Musicae Scientiae.*	Interviews with musicians (UK *n* = 8, Italy *n* = 9)	Qualitative design (semi-structured individual interviews)	Musicians´ experiences of playing in paediatric hospitals	To explore the motivations of musicians performing in health care settings to undertake their work, to explore the nature of their profession and professional identity	Musicians want to distract the audience/patients from a threatening environment, to provide them with a positive experience that will relax them, but also: change of emotional state, release of emotions although denying therapeutic aims but thought that music had positive consequences for patient´s wellbeing. Need of artistic competencies and abilities to respond to different musical styles and genres, evaluate the musical level of the performing patients. Professional identity based on personal motivations, musical and interpersonal skills despite the self-non-identification as “musician in hospital” as a profession (lack of formally sanctioned, representative professional body of knowledge in contrast to Music Therapy
13) C. Preti and G. Welch (2013)/Italy. **The inherent challenges in creative musical performance in a paediatric hospital setting**. *Psychology of Music.*	Interviews and surveys with musicians (*n* = 8)	Qualitative design (semi-structured interviews + self-reported measures), grounded theory	Investigate musicians´ experiences of performing for a non-voluntary audience (e.g., in paediatric hospital)	Identifying the inherent challenges of being a performer in a hospital, identifying the professional characteristics of the musicians who perform in such a setting	Risk of burnout—emotional and physical challenges, heavy workload—performing at a high standard, detecting the emotional situation, selecting the right piece of music to establish contact, perform variations/improvising. They are not equipped with either the emotional skills or musical techniques to cope with situations, relying on personal craft knowledge and experience rather than from the hospital training course. Potential importance of supportive supervision, no space for verbalizing experiences neither by their professional association nor by the hospital. No time for rehearsal of repertoire/sharing of materials. Although highly rewarded emotionally, not sufficiently aware of their professional identity, little sense of acting as a part of a collective
14) Preti and Welch (2012)/Italy. **The incidental impact of music on hospital staff: An Italian case study**. *Arts & Health.*	Interviews with hospital staff (*n* = 20) – (nurses (*n* = 11) doctors (*n* = 9))	Qualitative design, semi- structured interviews, Grounded theory	Discussions about experiences from musicians playing in paediatric hospital	To investigate the long-term effects of a live music programme on nurses and hospital staff and perceptions of how their long term exposure to a live music programme impacted (if at all) on their daily work? The staff´s awareness of any particular benefits of the music to their child patients? if such awareness helped them mediate between their individual liking of the music programme and the perceived impacts on the child-patients?	Life Music is reported as: stressful element, sameness of style, not so nice to have in background but generally accepted when directed at the children, help children familiarize, important with known repertoire, interaction, and improvisation. Musicians not perceived as professionals due to their way of working. An inability to plan for interventions together. Overall: Musicians in hospital- a professional status and need for communication skills. Need for cooperative guidelines to understand the elements that constitute a successful intervention.
15) Preti and Welch (2011)/Italy. **Music in a hospital: The Impact of a Live Music Program on Pediatric Patients and their Caregivers**. *Music and Medicine.*	Interviews with: Children (*n* = 14) Caregivers (*n* = 22) Observations on: Children (*n* = 162) caregivers (*n* = 146), musicians (*n* = 9)	Qualitative design (observations, audio-and video-recordings, interviews, fieldnotes)	Musicians playing in paediatric hospital, live music with musical interaction, familiar songs from a broad and flexible repertoire	Quality of interactions happening between musician, child and caregivers. Determine what kind of musical event was triggering a particular response an any of the groups involved.	Music is a powerful tool for alleviating some of the more negative aspects of hospitalization, nurturing a sense of individual, group, and institutional wellbeing, distract from stress, anxiety Familiarity of repertoire, use of percussion instruments are necessary tools to keep the children engaged. The mediation of the parent seems to be an important step towards establishing a musical channel between the musician and the child. Musical modalities and improvisation techniques is important. Music as a learning experience (both music and the self), engage cognitive skills, parents learning to use music for their children coping with stress and establishing relation. Neuroscience: the multi-sited nature of human musical behaviour.
16) Scheufler et al. (2021)/US. **Comparing Three Music Interventions for Anxiety and Relaxation in Youth with Amplified Pain**. *Journal of Music Therapy.*	Children patients (*n* = 48)	Quantitative (Quasi-randomized) design: VAS for Relaxation, State-level anxiety scale, pain, and demographic characteristics	3 weeks, 3 treatment crossover design with 3 interventions delivered in quasi-randomized order: 45 minutes of live patient-selected music (LPSM)/active music engagement (AME)/music-assisted relaxation (MAR)	To determine and compare the effectiveness of three well-established music therapy interventions: live patient- selected music (LPSM), active music engagement (AME), and music-assisted relaxation (MAR) on relaxation levels and state-anxiety in youth with APS (Amplified Pain Syndrome). Hypothesis: All three interventions would lead to significant improvement in relaxation, in state-level somatic symptoms of anxiety, and in state level cognitive symptoms of anxiety. The MAR-intervention (which is relaxation-focused) would have a greater effect on participant relaxation and somatic anxiety symptoms, whereas the AME and LPSM interventions (which are more focused on engaging with music) would have a greater effect on cognitive anxiety symptoms. There would not be an effect of number of sessions on these outcomes and there would not be an interaction between intervention and the number of sessions a youth had experienced.	1.Music interventions can lead to significant improvements in relaxation and state-level anxiety. All interventions had very large effects on the outcome of relaxation; very large effects were observed for the effect of LPSM on somatic anxiety and for AME on cognitive anxiety. No reported adverse effects for any of the three music interventions. 2. MAR intervention leads to a greater reduction in self-reported relaxation and to a greater reduction in somatic anxiety levels, all interventions led to similar changes in cognitive anxiety = not consistent with hypothesis. 3. Significant effect of week on self-reported relaxation but not on the other scales.
17) Sundar et al. (2016)/India. **Live Music Therapy as an Active Focus of Attention for Pain and Behavioural Symptoms of Distress During Pediatric Immunization**. *Clinical Paediatrics.*	Children Patients (*n* = 100)	Quantitative design/experiment group (*n* = 50), control group (*n* = 50) Modified Behaviour Pain Scale (MBPS) of children/Blood pressure, heart rates of parents.	Music therapy intervention in experiment group: singing and musical instrument playing along with visual aids (hand puppets and finger puppets) during the procedure of immunization.	To record the effect of music therapy interventions on behavioural changes during immunization on children and parents.	Live music therapy reduces duration of crying spells, improves pain perception, and reduces distress levels in children undergoing painful immunization procedures. Blood pressure and heart rate of the parent holding the child during the immunization procedure are not significantly affected.
18) Uggla et al. (2018)/Sweden **Music Therapy supported the health-related quality of life for children undergoing haematopoietic stem cell transplants**. *Acta Paediatrica.*	Children patients (*n* = 29 ( inpatient (*n* = 14), outpatient (*n* = 15))	Quantitative design (RCT, pilot study, Health-related quality of life (HRQoL), pain, mood—Paediatric Quality of Life Inventory 4.0 generic core scales, pre- and post-test. Crossover-design between groups	2× 4–6 Individual music therapy sessions (45 min) In hospitalization, or after discharge	Can music therapy reduce anxiety, improve mood, support the mental health recovery, and influence the rate and degree of physical recovery after allogeneic HSCT.	The combination of reduced heart rate values four to eight hours after the intervention in the music therapy group and the improved HRQoL reported by both groups suggest that music therapy can be an effective, complementary intervention during and after HSCT.
19) Uggla et al. (2016)/Sweden. **Music therapy can lower the heart rates of severely sick children**. *Acta Paediatrica.*	Children patients, age 0–16 (*n* = 21).	Quantitative design/randomized clinical pilot study, Disease severity (Lansky Play Performance Scale), Nutritional status, biological parameters (weight and Blood values), Heart rate, Blood pressure, blood value and saturation morning and evening.	Treatment group (*n* = 12) Music therapy (45 minutes), expressive and receptive (Creative and Free Improvisation—singing, playing, listening) twice a week. Control group (*n* = 9) standard care	To explore whether music therapy as a psychosocial intervention, could be evaluated by psychological parameters in a larger randomized setting of children undergoing HSCT, by comparing objective measurements in both the mornings and the evenings.	No significant differences in saturation levels between morning and evening. No significant differences in blood pressure between groups. In the music therapy group, the children´s heart rate decreased during the day—significantly lower for the music therapy group compared to the controls. Lowering heart rate = reducing stress levels for at least 4–8 hours.
20) Uggla et al. (2019)/Sweden. **An Explorative Study of Qualities in Interactive Processes with Children and Their Parents in Music Therapy during and after Pediatric Haematopoietic Stem Cell Transplantation**. *Medicines.*	Children patients with families (*n* = 6)	Qualitative design/collaborative research Interviews with families during and after Music Therapy intervention	Music Therapy intervention group: expressive and receptive method, twice a week for 4–6 weeks during donor. Control group: Music therapy after discharge twice a week for 4–6 weeks.	To investigate the subjective experiences and memories of interactions between children, parents, and a music therapist during music therapy interventions. The hypothesis of the study is that it is possible to identify important components and potential common threads in these interactions.	For the participants, music therapy developed into a significant and helpful experience. In this experience, the relationship and collaboration with the music therapist through meeting and playing together became a significant ingredient in coping with and managing the treatment period at the hospital. The children emerge as individuals taking initiative in the relational process, the importance of the parents as witnesses and helpers to their children.
21) Văduva and Balla (2019)./Romania. **A study of the efficiency of music therapy, art and play therapy on hospitalized children diagnosed with chronic illnesses**. *Studia UBB Musica.*	Children patients (*n* = 322), Group sessions = 302 with chronic progressive illnesses, individual sessions *n* = 20 in oncology and palliative care	Quantitative experimental design/SPSS Music therapy group (*n* = 191) Play and Art therapy group (*n* = 131) Observation sheets: *Facial expression* (gaze, level of brightness in the eyes, corners of lips up or down, position of head) *Behaviour* (body position—avoidance, acceptance, embrace, language, motor behaviour *Emotions* (joy, tranquillity, agitation, anger, fear, disappointment − 1–5 very low-very high) Observation before and after intervention from 47 sessions	Music intervention with Nordoff-Robbins model of creative improvisation and composition/receptive techniques Art therapy—creating an art project in group Play therapy—four stations of play activities—individual and socialization skills	To highlight the impact of alternative therapies on the emotional wellbeing of hospitalized children with chronic illnesses. Monitoring the facial expression, emotional disposition, and behaviour of hospitalized children before and after the music, play, and art therapy sessions. The role of these alternative therapies is to reduce tension, anxiety, and fear in hospitalized children and to help them manage their negative emotions. To analyse if there is a difference in the effectiveness of music therapy by comparison to play and art therapy as indicated in some research.	All therapeutic interventions were successful in elevating the level of perceived happiness. All alternative therapies are shown to have a beneficial impact in reducing anxiety and increasing the level of joy
22) van der Heijden et al. (2018)/NL **Can live music therapy reduce distress and pain in children with burns after wound care procedures? A randomized controlled trial**. *Burns.*	Children patients, age 1–3 (*n* = 135)	Qualitative design/RCT -study/Intervention group (*n* = 71), Control group (*n* = 64), video-observing level of distress assessed with Observational Scale of Behavioural Distress-revised (OSBD-r) + level of pain assessed with COMFORT-behavioural scale (COMFORT-B) before, walking to, walking into treatment and after. Children>5 years scored distress and pain on the validated Wong-Baker Scale (FACES) and Face Pain Scale-Revised (FPS-R.)	Intervention group: matched music intervention from MT of 3–5 minutes, Mother feeding and holding child. Control group: Standard care	To determine whether live music therapy directly after WCP could be beneficial in reducing children´s distress and pain. Hypothesis: 75% of the children in the intervention group would show less distress and pain after WCP compared to children in the control group.	Those in the music group (Children<5 years old) reported statistically significant less distress on the self-reported FACES scale than those in the control group. Effectiveness of live music therapy to reduce distress and pain associated with painful burn wound care was not shown in young children.
23) Wong et al. (2021)/Singapore **The Role of Music Therapy for Children Undergoing Cancer Treatment in Singapore**. *Healthcare.*	Children patients (*n* = 25)	Quantitative observational study—The Goal Attainment Scale (GAS) scored every 3 months	Individual personalized music therapy (receptive, recreative, improvisational, compositional)	To explore the benefits of MT for children with cancer. Describe profile and common goals of children requiring MT. To examine (a) the profile of patients who were referred for MT (b)frequency of accessing MT over a child´s cancer treatment (c) goals and objectives of MT services for children undergoing cancer treatment (d) efficacy of MT based on goals achieved.	Music therapy is an accessible and effective intervention that has therapeutic versatility in supporting functional and emotional goals. The most common need- the regulation of the child´s mood and morale. MT has the potential in addressing functional and psychosocial challenges for children undergoing cancer treatment.
24) Yates et al. (2018)/US. **Caregiver Perceptions of Music Therapy for Children Hospitalized for a Blood and Transplant: An Interpretivist Investigation**. *Global Advances in Health and Medicine.*	Caregivers of 14 Children patients (*n* = 15)	Qualitative design/semi structured individual interviews by Phone after children received music therapy	Individual Music Therapy sessions (cognitive behavioural approaches—active music engagement through instrument play, music for physical engagement, music for relaxation, instrument lessons and musical books) for 15–45 minutes twice a week until discharge. Caregivers were included.	To explore primary caregivers´ perspectives and experiences with music therapy for their children during hospitalization for BMT: What are caregivers´ perspectives and experiences with music therapy for their child hospitalized for BMT?	There are mutual caregiver and child benefits from music therapy after BMT. Music therapy can be a beneficial psychosocial treatment modality for all paediatric BMT recipients regardless of age or underlying diagnosis. Music Therapy can minimize the effect of isolation, negative mood, decreased activity, and enhancing normalization of childhood experiences during BMT recovery. MT promote both the caregiver and child´s psychosocial wellbeing.

**Table III.** Summary of quality appraisal after using MetaQuat tool.

Quality assessment	Yes	No	Un-clear	N/A
***1.Relevancy*** Does the study address a topic(s) relevant to the issue under investigation? *Was the justification for the study clearly stated? (For example, does it address a gap in the existing literature?), Do the results of the study apply to the issue under consideration? How similar or different is the study population or setting to yours? Is a difference likely to matter for the issue at hand? Is the research design appropriate for the methodology you are considering?*	22		2	
***2.******Reliability*** a) Is the study presented clearly? *Is the rationale for the study clearly stated, and does the study focus on clearly defined issue? Is the conduct of the study clearly described and easy to follow? Can you identify the research design? Are all relevant results included? Are the findings presented and discussed within the appropriate context? Is there a conflict-of-interest statement? Can the study be reproduced with the information provided?*	21		3	
b) Are the research methodology and results clearly described? *Does the methodology describe the population studied, the intervention given, and the outcomes? Are all sources of information clearly identified? Are inclusion and exclusion criteria defined? Are the statistical and/or analytical methods described? If applicable, are the results reported in data tables consistent with those described in the result section? Could the methods be reproduced on the information provided?*	19	1	4	
c) Are ethics procedures described? *Was appropriate informed consent obtained? Was the study approved by an ethics review board?*	20	2	2	
***3. Validity*** a) Is the study methodology appropriate for the scope of research? *Is the research question congruent with the research design? Does the methodology match the theory or the conceptual model? Are appropriate controls considered if applicable? Are the statistical/analytic methods appropriate for the design and/or the question? Are important theoretical factors accounted for in the analysis?*	20	2	2	
b) Is the research methodology free from bias? *Were there major sources of bias with respect to study design? … study participants inclusion/exclusion? … measurement of exposure/outcome or important confounders/predictors? … data sources? … analysis? … selection of studies? Are all comprehensive factors included in the research? (i.e., were important factors not measured that are critical to interpretation?) Are the results consistent within the study? Can chance findings be ruled out? Were the analyses carried out appropriately?*	12	3	9	
c) Are the authors´ conclusions explicit and transparent? *Are the results conclusive? Are the authors´ conclusions clearly derived from the results (transparent)? Are potential discrepancies discussed?*	16	4	4	
d) Can I be confident about the findings? *Are there any major methodological flaws that limit the validity of the findings (these may have been identified in a) or b))? Is the study´s result similar to those of the existing body of literature? If not, are the reasons for the difference clearly explained?*	16	3	5	
***4. Applicability*** Can results be applied within the scope of public health? *Can the study´s results be interpreted and analysed within the context of public health? Are there other important public health outcomes to be considered that were not included?* *Can the results be applied to public health practice, based on the validity of the article and its relevance? Are harms and benefits discussed? Were the relevant stakeholders considered?*	19		5	
***5. Summarized quality of 9/9 possible items of Y/N/UC/NA*** *Y/T ≥ 75% or N+U/T ≤ 25% = High/good quality* *(100% = High, 75–99% Good, 50–75% Low, 25–49% Very low)*	**High** 11	**Good** 5	**Low** 3	**Very** **Low** 5

Through the analysis, four quantitative and three qualitative categories were outlined. Features from the charting of quantitative data may additionally be perceived to support the interpretation of qualitative categories. Quantitative data were charted into the categories: *Included studies—design and methodology*, *Populations, Live music intervention—design and profession in charge* and *Intervention objectives in relation to health and wellbeing*. Qualitative codes and themes were categorized through: *Impacts qualitatively elaborated, Subgroup perspectives* and *Characteristics—promising aspects, barriers, and facilitators*. Following guidelines for reporting music-based intervention by Robb et al. (2011) considering “the variety, complexity, and uniqueness of such interventions” (p. 342), we understand that clear descriptions of theory, content, delivery schedule, interventionist, treatment fidelity, setting, and unit of delivery “are essential to improve replication and translation of music-based interventions to clinical practice” (p. 348). These recommendations will further guide our reflections.

## Results

First, the results are based on 24 articles of different designs, see Table II. To begin with, the four quantitative categories showing countable features in relation to research questions 1–5 are presented. These are followed by three interpretive qualitative categories consisting of outcomes in accordance with research questions 5–8. Finally, the last category of quality appraisal responding to research question 9 is presented together with a synthesis of results.

## Included studies—design and methodology

Eight of the included studies were from Italy (Antonelli et al., 2019; C. Preti & G. F. Welch, 2013; C. Preti & G. Welch, 2013; Giordano et al., 2020, 2021; Longhi et al., 2015; Preti & Welch, 2011, 2012), six from the US (Bush et al., 2021; Colwell et al., 2013; Millett & Gooding, 2018; Ortiz et al., 2019; Scheufler et al., 2021; Yates et al., 2018), three from Sweden (Uggla et al., 2016, 2018, 2019), two from the UK (Blackburn, 2020a, 2020b), and one study each from India (Sundar et al., 2016), Romania (Văduva & Balla, 2019), the Netherlands (van der Heijden et al., 2018), Singapore (Wong et al., 2021) and Austria (Grebosz-Haring & Thun-Hohenstein, 2018). More than half of the studies (*n* = 13) had a quantitative design (Antonelli et al., 2019; Bush et al., 2021; Giordano et al., 2020, 2021; Grebosz-Haring & Thun-Hohenstein, 2018; Longhi et al., 2015; Millett & Gooding, 2018; Scheufler et al., 2021; Sundar et al., 2016; Uggla et al., 2016, 2018; Văduva & Balla, 2019; Wong et al., 2021). Eight of these studies used an RCT or other experimental design. In quantitative studies, assessments were made by either biological measures or validated scales screening features for pain perception, mood, anxiety, and attention. Ten studies had a qualitative design (Blackburn, 2020a, 2020b; C. Preti & G. F. Welch, 2013; C. Preti & G. Welch, 2013; Colwell et al., 2013; Preti & Welch, 2011, 2012; Uggla et al., 2019; van der Heijden et al., 2018; Yates et al., 2018) with validations preferably from semi-structured interviews and observations. Only one study exhibited a mixed-method design (Ortiz et al., 2019), using both validated measures, observations and open-ended questionnaires.

## Populations

In 17 of 24 eligible studies (71%), *children and young people* were the core population studied. In total, 1,035 children/young people participated in these 17 studies, where a majority encountered a live music intervention. However, children were sometimes randomized to control groups with standard care where no musical activity was offered. Ages varied between 0 and 17 years. However, eight of 17 studies neither defined the age of participating children nor reflected on issues of age. Only two studies defined an examination of outcomes for toddlers, young children 0–3 years old, while the seven remaining studies reported different age ranges for the child patients. Most studies in the population, children, and young people (12/17, 70%), had a sample of between 10 and 50 participants. One study had a sample of less than 10, whereas four studies had samples of more than 100.

In the 17 studies of children and young people, three broader areas of population diagnosis and treatments were reported: *Preoperative care* – invasive procedures, haematopoietic stem cell transplant (HSCT), immunization, wound care procedures, preoperative anxiety (9/17, 53%), *General paediatric issues* - hospitalization, amplified pain, mental disorder (5/17, 30%), and *Emergency care* – traumatic brain injury, mechanical ventilation, and sedation, cardiac- and/or respiratory problems (3/17, 17%). *Families* were represented in seven studies (7/24, 29%); hence, most of those studies were also reported within the population of children/young people. Only three studies (3/24, 12%) described families’ perspectives exclusively (Ortiz et al., 2019; Preti & Welch, 2011; Yates et al., 2018) although Preti and Welch (2011) also included perceptions from the child patient population. *Health care professionals* were the core population in only two (2/24, 8%) studies (Blackburn, 2020b; Preti & Welch, 2012), examining their perspectives on outcomes from live music interventions. However, health care professionals were briefly represented in Antonelli et al. (2019) and Giordano et al. (2020). Finally, two (2/24, 8%) of the included studies explicitly presented the perspectives from *health musicians* working in paediatric hospitals (C. Preti & G. F. Welch, 2013; C. Preti & G. Welch, 2013).

## Live music interventions—design and profession in charge

In most studies (21/24, 87%), the musical interventions targeted individual children, singing and playing together, preferably at the bedside. Here, a board-certified music therapist made most of the interventions (13/21), often individually tailoring activities to fit the state and condition of the child. However, some individual interventions (4/21) were led by professional musicians while playing at the bedside, inviting children to participate. In the remaining studies (4/21) describing individual interventions, the profession in charge was not defined. Two studies (2/24, 8%) presented participatory singing games led by vocal tutors, and group music intervention was described only in one study (1/24, 4%) led by a musician, although hospital musicians, while working at bedsides, in wards and waiting rooms, occasionally met groups of children and families. In most studies (14/24, 58%), the intervention timeframe was not defined, although in those where it was, 45 min of delivery was the most common (5/24, 21%). The five remaining studies presented intervention timeframes from 3 to 45 minutes, on average 5–15 min. In more than half of the studies (13/24, 54%), assessment of outcomes was made following a single intervention, whereas the remaining studies presented designs with 4–6 or more interventions in a row. The most common ways of collecting data (both quantitative and qualitative material) connected to interventions were both pre- and post-testing, or just post-testing (22/24, 92%). One study collected data during and after intervention, while another did so pre-, during and post-intervention. Only one of those studies followed subsequent interventions and outcomes for more than three months, in some cases up to one year (Wong et al., 2021).

## Intervention objectives and impacts in relation to health and wellbeing

While quantitatively screening all 24 included studies for defined objectives and impacts, three themes were outlined containing different features of health and wellbeing that had been examined empirically. Most studies presented features from more than one of those themes as they are clearly interconnected. Firstly, outcomes of *positive affect* means modifying affects and mood levels, reducing anxiety, fear, and distress. Within this theme, sedation and relaxation were expected to increase with the help of the musical interventions offered. The second theme, *coping*, includes distraction, psycho-social engagement, decrease of pain perception, and better pain management in an unfamiliar and often frightening environment. In the third theme, *reducing hospitalization*, the live music intervention was deemed to support participation, agency, child rights and the overall wellbeing of the child patient. Although included studies exhibit a variety of designs and data collection methods, these themes extensively cover all collected objectives and impacts studied.

## Impacts qualitatively elaborated

Codes and themes from the qualitative content analysis were sorted according to the research questions. Thus, impacts and outcomes could be reflected through more interpretative features, although quantitatively displayed themes of intervention objectives (positive affect, coping, reducing hospitalization) showed themselves to be a useful frame for presentation. These features were further examined through subgroup perspectives. When qualitative codes and themes were elaborated and compared, interrelated prerequisites, such as promising aspects, barriers, and facilitators could be seen.

### Positive affect

The impact of positive affect is perceived as reducing anxiety, fear, and distress as well as modifying affects and mood levels. Among the included studies, this impact is the most referred to, although features are both quantitatively and qualitatively measured. For example, Wong et al. (2021) suggests that the most common need is the regulation of the child’s mood and morale. When comparing three non-pharmacological live interventions in an Italian emergency care unit, the analysis did not show any difference for live music interventions led by professional musicians compared to interventions with dogs or clowns (Antonelli et al., 2019). The core findings still suggested that all activities empowered *positive affect* (e.g., happiness, excitement, energy) in a similar way, interpreted by authors as important measures of increased wellbeing despite the children’s experiences of distress. In other studies, music interventions showed more specific results. While supporting positive experiences and reducing negative emotions, music interventions increased positive affect and helped distract the child (and family) from the unfamiliar hospital environment and frightening treatments (Blackburn, 2020a, 2020b; Preti & Welch, 2011; Uggla et al., 2018).

Several studies interpreted physiological measures as vital signs of decreased stress (e.g., decreased heart rate levels, respiratory rates, mean arterial pressure and salivary cortisol) (Bush et al., 2021; Grebosz-Haring & Thun-Hohenstein, 2018; Longhi et al., 2015; Sundar et al., 2016; Uggla et al., 2016). For example, decreased heart rate, even four to 8 hours after an intervention, was interpreted as improving the health-related quality of life (HRQoL) (Uggla et al., 2018) in the categories anxiety, mood, mental and physical health. Hence, outcomes of positive affect are understood to influence both psychological and physiological parameters (Uggla et al., 2016). Also, based on observations and self-reports, live music therapy was reported to reduce emotional distress and anxiety, and to support sedation (Giordano et al., 2020, 2021; Millett & Gooding, 2018; Ortiz et al., 2019; Scheufler et al., 2021; van der Heijden et al., 2018), to decrease the intensity of agitation and fear and to increase both peacefulness and the intensity of joy (Văduva & Balla, 2019). While elevating mood, musical activities also motivate the patients to physically engage (Yates et al., 2018). In contrast, Colwell et al. (2013) did not find any clinically significant changes in heart rate, blood pressure, or oxygen saturation after an intervention, albeit self-reported anxiety was still reported to decrease. Findings from semi-structured interviews with children, families, healthcare professionals and musicians in charge also displayed outcomes of modified affect coupled to decreased stress levels and relaxation (C. Preti & G. F. Welch, 2013; C. Preti & G. Welch, 2013; Preti & Welch, 2011, 2012)

### Coping

In the studies, features involved in modifying affect are consistently discussed as enhancing for the ability of the child and family to *cope* with symptoms and/or emotions related to medical procedures, and other demanding or frightening aspects of paediatric hospital care. Pain perception and/or pain management, are one recurrent theme. Longhi et al. (2015) showed significant decreases in heart rate and pain level after music sessions compared to reading/no interaction, arguing that the live music intervention impacts on both relaxation and pain distraction. Similarly, Scheufler et al. (2021), albeit comparing three different live music therapy interventions, AME (active music engagement), LPSM (listening patient selected music), MAR (music assisted relaxation) still found music activities to have an impact on anxiety and relaxation levels across all interventions while “decreasing the impact of pain by improving stress and pain management strategies and restoration of function” (p. 195). Ortiz et al. (2019) also showed that preparation and collaboration between “Child life and music therapy intervention supported healthy, adaptive coping and helped minimize distress experienced by patients during IV placements” (p. 1). For children (and caregivers), painful procedures can often be perceived as worse than the actual symptoms, whereas coping with distress and pain are at the fore in many of the studies. For example, Sundar et al. (2016) propose that live music therapy offered during painful procedures reduces duration of crying spells, improves pain management, and reduces distress levels in children. Music therapy is perceived as a helpful experience and plays an important part in the development of coping strategies, supporting the child to manage the treatment period at the hospital (Uggla et al., 2019). In Colwell et al. (2013), some coping strategies are displayed as “positive facial affect, active engagement (participation) and initiation, eye contact, and verbal interaction” (p. 255). Music interventions are reported to help distract children and families and focus their attention on something external to illness, while supporting psychosocial behaviour (C. Preti & G. F. Welch, 2013; Preti & Welch, 2011; Wong et al., 2021), emphasized as one very important coping strategy (Colwell et al., 2013). There are still discrepant findings concerning coping related to pain management regarding age. Live music therapy interventions did not show effectiveness in reducing distress and pain in young children after painful procedures (burn wound care), whereas older children, also more responsive to interventions, showed a significant decrease in self-reported pain (van der Heijden et al., 2018). This was also in line with findings from Antonelli et al. (2019), showing significant decreases in self-reported pain and positive affect after intervention, but only in older patients.

### Reducing hospitalization

Quite a few of the included studies come to the same conclusion, the importance of *reducing hospitalization*. This entails reducing negative experiences and the enhancement of coping strategies, which in turn both include, and preclude engagement and participation of the child patient. It is often a question of redirecting patients from pain, nausea, fatigue, or unpleasant procedures by creating a calm and engaging environment (Yates et al., 2018) or providing distraction and entertainment to increase joy and self-esteem (Văduva & Balla, 2019). Such features are reported to be closely connected to overall well-being, reducing the negative effects of hospitalization. For example, Blackburn (2020a) suggests that experiences from live music interventions while offering distraction from distress, restore the child´s control and improve the whole hospital experience. Similarly, Ortiz et al. (2019) proposes that music therapy interventions while enhancing coping and reducing anxiety also improve the child’s resilience and autonomy. Findings from Blackburn (2020b) suggest that participants in live musical activities highly value the human connectedness, which, by distracting them from hospital circumstances, cheers up, activates, and gives the children choice, whilst at the same time promoting both motor- and neurodevelopment. The authors further indicate the importance of play as an ingredient in music activities to promote both a sense of normality and the creation of meaning (Blackburn, 2020a). Together with a developmental stimulation, such features may “decrease immediate and lasting negative emotional impact of treatment” (p. 7). While focusing attention on something external to illness, the psychosocial space gives the child and the family an occasion to interact, which alleviates negative aspects of hospitalization and nurtures wellbeing (C. Preti & G. F. Welch, 2013; Preti & Welch, 2011). The psychosocial space offers a normalizing opportunity for childhood experience and gives mutual caregiver and child benefits regardless of age and diagnosis (Yates et al., 2018). For children with severe illnesses, music therapy interactions may represent a break in monotonous isolation and preserve a connection to life outside the hospital, offering a sense of being alive (Uggla et al., 2019; Yates et al., 2018). By the activation of positive emotions (and bodily sensations) such as curiosity, joy, liveliness, and energy arousal, the child can experience a diversion from pain and fear and feelings of competency and self-assertion (Uggla et al., 2019, p. 5). Through opportunities for emotional regulation, live music therapy is said to support the expression of thoughts and emotions, giving the child patient a better chance to emerge from treatment without being traumatized, potentially preventing the development of PTSD (Uggla et al., 2016).

## Subgroup perspective

### Musicians and music therapists

In the screening, few studies were found with the core objective to represent the professionals who perform the live music interventions. Only two studies exhibited a core focus (C. Preti & G. F. Welch, 2013; C. Preti & G. Welch, 2013), while a few other texts presented some professional considerations mainly from a music therapist perspective (Bush et al., 2021; Colwell et al., 2013; Giordano et al., 2020, 2021; Ortiz et al., 2019; Scheufler et al., 2021; Uggla et al., 2016, 2018, 2019). The musicians represented in the two core studies were from Italy and the UK and were hired by either a charity or a special hospital programme. All musicians included were concurrently playing in orchestras or had other freelance commitments alongside working in paediatric healthcare Findings show that to play for children in hospitals is consistently perceived as emotionally rewarding, with both moral and religious reasons for carrying out the work (C. Preti & G. F. Welch, 2013). While discussing important outcomes for children and family, informants reported that the live music interventions may relax and distract both patients and parents from a threatening environment. In both studies, specific professional skills, reaching beyond their core artistic competence, are perceived as prerequisites to manage the work. Included here are “social interaction, empathy, appreciation, openness, flexibility and humour” (C. Preti & G. F. Welch, 2013, p. 359) but also a large portion of musical improvisation skills (C. Preti & G. Welch, 2013). According to C. Preti and G. F. Welch (2013), the audience in paediatric hospital care is often in distress and pain, which can create musical, emotional and physical challenges for musicians who are not sufficiently trained for such encounters. Despite being emotionally rewarding, work can thus create a health cost for music professionals, and the authors conclude that there is a need for a professional organization to support this emerging profession, where supervision, peer reflection and time for rehearsing ought to be included (C. Preti & G. Welch, 2013). Other professional prerequisites communicated in the studies are the importance of a trained music therapist who may adapt and tailor the intervention to fit and follow the child’s mood and condition (Bush et al., 2021; Colwell et al., 2013; Giordano et al., 2020, 2021; Scheufler et al., 2021). Another recommendation, above all connected to the music therapy profession, is to build a therapeutic relation with the child, which is sensitive to culture and family relations (Ortiz et al., 2019; Uggla et al., 2016, 2018, 2019).

### Family

Caregiver perspectives are clearly interwoven in some of the studies, whereas three studies have this population at the fore (Ortiz et al., 2019; Preti & Welch, 2011; Yates et al., 2018). Depending on different methodological implementations, studies report heterogeneous findings, showing musical interventions having impacts on family members. Giordano et al. (2021), compared the level of anxiety and sedation in paediatric patients undergoing invasive procedures with or without the presence of music therapy intervention, showing a positive impact from the live music intervention. In this study, the mothers´ anxiety level was assessed along with their child’s, and it was found that there was an equal effect on mothers, also having a positive impact on the mother-child dyad. Similarly, Millett and Gooding (2018) propose that the music therapy intervention affects caregiver anxiety. Thus, music therapy activity is advocated as an “effective patient- and family-centred treatment” (p. 473). In Ortiz et al. (2017) parents´ perceptions of positive impacts from music interventions on their children show that the collaboration between child life and live music intervention may prepare and support healthy, adaptive coping while minimizing distress experienced by child patients during intravenous placement. Positive outcomes are perceived to be due to the therapeutic alliance with both child and family. Although parents are often worried and protective in hospital situations, they are important interpreters of their child’s behaviour and significant assistants for musicians when successfully monitoring the music intervention (Preti & Welch, 2011). One beneficial impact may even be that parents learn the repertoire used and gain new tools for distracting their child on their own (p. 219). Another reported beneficial impact is parents´ appreciation of seeing their child happy, engaged, and participating, which provided a space for relief, peace, and comfort also for them (Blackburn, 2020b; Yates et al., 2018). A subsequently reported caregiver anxiety, paired with a clear and understandable worry aimed at protecting the child from harm (Preti & Welch, 2011), might explain why parents reported lesser effects on themselves although child patients felt lower levels of anxiety and pain (Longhi et al., 2015), or that parents reported lower HRQoL for their children than the child’s self-reported HRQoL (Uggla et al., 2018). In line with this, results from Sundar et al. (2016) showed that blood pressure and heart rate of parents holding their child during immunization procedures accompanied by music intervention, were not significantly affected.

### Health care professionals

Compared to the more common establishment of music therapists in paediatric health care in many countries, albeit increasing in numbers (Preti & Welch, 2011), professional health musicians are still not currently found in these settings. The conceptual clarification and professional expectations of health care musicians compared to music therapists is equally not yet outlined (Koivisto & Tähti, 2020). There is currently a lack of formal education in music, health and well-being for nurses and doctors (Preti & Welch, 2012). While musicians in the collected studies target suitable children on a more random level, both at the bedside and on wards (albeit in collaboration with staff), health care professionals report that both the presence of the musicians and the acoustics of the music sometimes interfere with care (Antonelli et al., 2019). Further, staff sometimes complain about the sameness of repertoire (C. Preti & G. Welch, 2013). Health care professionals interviewed in Preti and Welch (2012) reported that the music performed could be either pleasurable or distracting for their work, often perceived as stressful in an already emotionally demanding work situation. At the same time, Longhi et al. (2015) found staff to be generally positive while perceiving the music as comforting the children. Again, according to Preti and Welch (2012), health care professionals still reported the music as helping their child patients to become familiar with the new hospital environment and make them much calmer in an emergency department. Nurses reported that they often used music themselves (radio or ringtones) to help distract the child and cope with procedures, showing improvisation to be a skill also for nurses. At the same time, due to a busy workload, they felt unable to plan for musicians to support children during painful procedures. From their point of view, the musicians are preferably not perceived as professional performers although they consider the most important role of the musicians is to engage musically with child and family (Preti & Welch, 2012). In other studies, while reflecting on the work of a music therapist, health care professionals say that the music interventions helped them to get to know their patients, to meet the emotional needs of children and family, and that it was encouraging to see the children enjoy themselves (Blackburn, 2020b). Similarly, both Ortiz et al. (2019) and Giordano et al. (2020) found music interventions to be satisfying and calming for staff. When children become more peaceful and collaborative due to the music activities, health care professionals feel more confident and serene in their own work (Giordano et al., 2020).

## Characteristics—promising aspects, barriers, and facilitators

By mapping impacts and subgroup perspectives together some characteristics emerge. To discriminate reported prerequisites for the implementation and execution of live music interventions, these results are presented below as conclusive subcategories of promising aspects, barriers, and facilitators with implications for practice.

### Emotional regulation

The child’s emotional state is monitored during live music interventions (Antonelli et al., 2019). This means that significant decreases in heart rate, respiratory rate and mean arterial pressure (MAP) are rated as physiological signs of decreased anxiety (Bush et al., 2021). Following this, the impact, often measured as reduced anxiety, reduced stress, and supported sedation points to live music intervention as an important complementary method for the emotional regulation of the child patient (Colwell et al., 2013; Giordano et al., 2021; Grebosz-Haring & Thun-Hohenstein, 2018; Uggla et al., 2018), even influencing the impact of the acoustic ecology of the hospital (Blackburn, 2020b). To focus emotional regulation through live music intervention displays a crucial characteristic for reducing preoperative anxiety (Giordano et al., 2020; Millett & Gooding, 2018), distracting and supporting both before and during painful procedures (Sundar et al., 2016; Văduva & Balla, 2019), supporting relaxation (Preti & Welch, 2011; Scheufler et al., 2021), and helping the child patient develop coping strategies, which in turn support pain-management, self-esteem, physical activation and psychosocial stimulation (Colwell et al., 2013; Scheufler et al., 2021; Uggla et al., 2019; Văduva & Balla, 2019). Interactive emotional regulation, through tailored musical interventions together with a trained music therapist, helps the child to express emotions and to achieve emotional experiences of safety, trust and belonging that also might prevent future treatment anxiety and traumatization (Uggla et al., 2016, 2019).

### Play and participation

Participation through opportunities for play and creativity within musical activities is said to be an important facilitator to promote a sense of control, self-esteem, and agency (coping) for the child patient (Blackburn, 2020a; Colwell et al., 2013). Further, according to Blackburn (2020b), participation through the child’s own choice is perceived as empowering. Activities bring a sense of normality in abnormal circumstances while creating positive relational experiences and good memories, also for parents (Blackburn, 2020b; Uggla et al., 2019). The presence of opportunities for connectedness and new relations in hospital care may even create a kind of “hospital family” which further helps to normalize the hospital experience (Blackburn, 2020a, 2020b). Giordano et al. (2020), while referring to Ghetti (2012), argue that musical interaction tailored by a trained music therapist offers the child a support, which contrasts the passivity of submitting oneself to treatment. Following Uggla et al. (2019), it is further argued that what may be offered through musical interaction will give the child patient an experience of being recognized. Recognition is made within an intersubjective field where meaning is shared (e.g., shared attention, shared intentionality, shared affectivity). Closely connected to play, the live music interactions may also facilitate non-verbal communication (Preti & Welch, 2011; Văduva & Balla, 2019) seen as highly experimental activities with body sensations and affects as essential components (Uggla et al., 2019). As play and creativity allow self-expression (Blackburn, 2020a) the child may experience meaningful achievements of bodily functions and activity participation, supporting both functional and emotional goals, which further promote both the caregiver and the child’s psychosocial wellbeing (Wong et al., 2021; Yates et al., 2018). In this child- and family-centred process, Uggla et al. (2019) highlight the importance of parents as both witnesses and helpers.

### Age

One barrier for impact seems connected to age. In Antonelli et al. (2019) and van der Heijden et al. (2018), only older children were responsive and presented higher scores in pain assessment, although Antonelli et al. (2019) report that younger children still showed more open pleasure from music interventions than their elder peers. According to Uggla et al. (2016), it is more important to focus on the parent-infant interaction and the opportunity for non-verbal communication with young children.

### Session design

Preparation, design, best moment, timeframe, and currency of the live music interventions are reported as both barriers to, and facilitators for, positive outcomes. Design seems to be most important, where children are offered participation through the music activities (Blackburn, 2020a, 2020b; Giordano et al., 2020; Preti & Welch, 2011; Uggla et al., 2016, 2018, 2019; Văduva & Balla, 2019; Wong et al., 2021). With musical improvisation, together with musicians, both child and parent can be engaged (Preti & Welch, 2011). At the same time, it seems to be important that each intervention provides structure and predictability that support both the child’s autonomous behaviour (through choice making and control) and involvement (Blackburn, 2020b; Scheufler et al., 2021). To succeed, Yates et al. (2018) identify the flexibility of music therapist as being most important. Following the findings from parents´ perceptions, it is recommended to schedule music interventions in paediatric care regularly to promote physical activity and mood (Yates et al., 2018). Similarly, Uggla et al. (2019) argue that the framework of implementing music therapy in paediatric hospital care is of great importance, which according to Ortiz et al. (2019)can constitute challenges regarding the coordination of care. This includes both the design and the number of sessions. Further, it is crucial to give enough time to develop a therapeutic relationship (attachment to therapist) to help the child handle emotions within a window of tolerance (Uggla et al., 2019). A number of sessions are highlighted in Preti and Welch (2011), where children during long hospital stays seem to become more and more familiar with the music activities. Millet et al. (2017) highlight the best timing for interventions to support the child in relation to aspects of surgery preparation and the often long delays in transfer time. One core facilitator highlighted the collaboration with staff prior to a musical project (Colwell et al., 2013), where a clear communication of aim, structure, and potential benefits could prepare and support more positive outcomes for child patients (C. Preti & G. Welch, 2013). Questions of best spaces for music and repertoire—also pleasing the staff audience—may thus be discussed with the staff. To support participation (for children who are not bed bound), the best space for music activities could sometimes be in waiting rooms where they can move more freely in the presence of other children (Preti & Welch, 2011). Finally, one facilitator supporting positive outcomes is the collaboration with other professionals targeting similar issues of wellbeing among the paediatric population. Ortiz et al. (2017) recommend cooperation between child life activities and tailored music therapy interventions to successfully prepare, and emotionally monitor, the child before and during painful procedures.

### Adaptivity and familiarity

In many of the included studies, adaptivity executed by the profession delivering music interventions is at the core (Bush et al., 2021). This means that one prerequisite for outcome is coupled to the musician/music therapist individually tailoring music activities and repertoire to immediate needs (Giordano et al., 2020, 2021; Scheufler et al., 2021; Yates et al., 2018) while matching “music characteristics with patient’s corresponding behavioural and physiological states” (Millett & Gooding, 2018, p. 473). This in turn requires improvisation in collaboration with both child and environment, including constant awareness paired with interpersonal skills regarding the selection of appropriate repertoire and offering practical choices for child participation (Preti & Welch, 2012). Again, arguing for a therapeutic relation, the music therapist adapts to the child’s musical preferences, energy, needs and physical state hence enabling the child to be present. Through such adaptivity, the child can stay emotionally regulated (Uggla et al., 2018). Person-oriented, music interventions may offer the patient a sense of control, a crucial component for vulnerable and seriously ill children (Uggla et al., 2016). Adversely, such multifaceted competence of decoding and reacting through empathy and intuition is reported to be demanding for the health musicians, while professionally negotiating their commission between distraction for wellbeing or therapy (C. Preti & G. F. Welch, 2013). Another important facilitator reported for positive outcomes is the use of familiar music (Blackburn, 2020a; Preti & Welch, 2011, 2012; Uggla et al., 2016) giving space for memories, routine, and connection to normal life (Blackburn, 2020a). In their study, van der Heijden et al. (2018) did not find music therapy effective in reducing distress and pain in young children after burn wound care. However, based on their study of very short and standardized music interventions of 3–5 min, the authors´ suggestion for future research is to focus on the parent–child dyad, as distressed infants tend to seek proximity to their caregiver, to adjust the interventions to the child’s need, and to consider the influence of timing for the intervention.

## Quality appraisal

Collected research is of heterogeneous design and methodology as well as covering a variety of objectives and reported impacts following live music interventions. Still, all findings could be charted around some conclusive features connected to health and wellbeing. Summarizing the nine quality appraisal criteria, most studies 16/24 (67%), were assessed as being of high, or medium quality, which means satisfactory scientific execution in the categories of relevancy, reliability, validity, and applicability. Spot checks of quality appraisal were made by the second and third authors, and discrepancies were discussed with the aim of reaching consensus. However, eight of the twenty-four studies (33%) were rated as low or extremely low quality (Table III). Following the template of the MetaQuat tool (Rosella et al., 2015), additional documents presenting the quality appraisal of each study can be requested from the authors. Due to discrepancies in scientific quality, some evidence could be argued to be questionable when drawing conclusions for practice. Some of these concerns will be reflected in the next section.

## Synthesis

The result section has presented findings from an analysis following the overarching research question: *In what ways are live music interventions and their outcomes described, assessed, and perceived to act as resources for health and wellbeing in children, families, and health care professionals in paediatric hospital care?* Although heterogeneous, all studies presented features connected to the study objective. In comparison, due to study design and methodology—some impact categories were perceived to be more dominant than others, while other categories presented some important prerequisites for reported outcomes. As an evaluation, a synthesis of quality appraisal has additionally been executed. In the following section, we will examine and expand some of these features in relation to theory.

## Discussion

The objective of this scoping review was to compile and examine characteristics and impacts of live music interventions on health and wellbeing for children, families, and health care professionals in paediatric hospital care. We chose a scoping review to examine settings, populations, and interventions in empirical research and to compare and evaluate reported results. As presented in the result section, when features of the presented impacts, i.e., *positive affect, coping* and *reducing hospitalization*, as well as *subgroup perspectives*, were examined and elaborated into crucial prerequisites, *emotional regulation* was displayed as a core promising category. Important facilitators of *adaptivity and familiarity* emerged here in relation to emotional regulation. Interrelated, but more practical, there were barriers and facilitators throughout the categories *play and participation, session design* and *age*. Charted in this way, both relational and practical implications seem equally important to support claims of health and wellbeing with the help of live music interventions in paediatric hospital care. Further, when perceiving music, health and wellbeing as ecological phenomena (Ansdell & DeNora, 2012; Elliott & Silverman, 2013) philosophical implications appear to be crucial while critically reflecting these findings. Firstly, we will reflect on collected findings in relation to the heterogeneity and assessed quality of study designs. Findings will then be discussed and elaborated within these three meta-categories defining philosophical, practical, and relational implications: *Music for wellbeing, Design for participation* and *Emotional regulation*. Secondly, we will reflect on the strength, limitations, and methodology of this scoping review. Finally, based on the elaboration of findings, a short conclusion with recommendations for practice and future research is presented.

## Quality appraisal and findings

Sixteen out of 24 studies showed high or good quality when assessing nine different items in the categories: relevancy, reliability, validity, and applicability. The often weak reporting concerned the presentation of design and methodology with additional effects on the question of bias. The lack of methodological transparency further affected the assessment of validity and applicability, and flaws were found in both quantitative and qualitative designs. Regarding applicability for practice, some impacts reported in this review could thus be questioned. Again, following Robb et al. (2011), we understand that clear descriptions of theory, content, delivery schedule, interventionist, treatment fidelity, setting, and unit of delivery “are essential to improve replication and translation of music-based interventions to clinical practice” (p. 348). Studies where such descriptions lack transparency weaken the validity of conclusions. For example, in studies of lower quality, claims of outcomes connected to music interventions lack at least one of the following items: descriptions of intervention protocol (e.g., content, repertoire, opportunities for child participation, theoretical foundation), timeframe for intervention, professional delivery and possible confounders (e.g., age, participating parents, medication) affecting results. With the lack of such descriptions, the live music intervention sometimes seems to be regarded as merely a sound object affecting the receiver, rather than an event where the distribution of communicative, intersubjective, and cultural features may afford emotional and social experiences (Elliott & Silverman, 2013; Malloch et al., 2012). For example, several studies included physiological measures as vital signs of decreased stress (e.g., decreased heart rate levels, respiratory rates, mean arterial pressure and salivary cortisol) (Bush et al., 2021; Grebosz-Haring & Thun-Hohenstein, 2018; Longhi et al., 2015; Sundar et al., 2016; Uggla et al., 2016), but reported impacts were considered either as directly affected by the music per se or as evolved from the situated distribution of an intersubjective intervention. Although collected research objectives and hypothesis were eligible for this review, we argue that depending on theoretical perspectives, the epistemological lenses used will determine the perception of outcomes and their implications.

## Music for wellbeing

Departing from contemporary conceptualizations of music, health, and wellbeing, the philosophical foundation of claims connected to *music for wellbeing* in collected studies may be scrutinized. Apart from the descriptions and assessments of impacts, we were especially interested in understanding how the music interventions were perceived to act as resources for health and wellbeing. In other words, which practical implications and interrelated theoretical conceptualizations could be traced. Perceiving music as a communicative and relational medium—psycho-biologically inherited although socially developed (Malloch et al., 2012) – makes music, and musical (inter)actions, a meaningful resource in the process of cultivating wellbeing (Ansdell & DeNora, 2012). Departing from an interpretation of music as related to social, cultural, and psychological variables, musical experiences might be understood as reaching beyond the perception of sounds, concurrently influencing affects, thoughts, and behaviours (Elliott & Silverman, 2013). If music is argued to be a resource for health and wellbeing in paediatric hospital care, different branches of interpretation may be used concurrently. The issue is how core concepts are elaborated and how conclusions are made. Following the WHO (2020), the definition of health clearly emphasizes a connection to physical, mental and social wellbeing, and not only the absence of disease and infirmity. This may in turn support critical reflections of study results claiming live music interventions to alleviate symptoms, while at the same time considering overall health and wellbeing as an objective. In some of the included studies, measurable physiological signs are understood as outcomes of positive affect. This is further connected to overall wellbeing but without a concern for *how* interventions are designed, performed, distributed, afforded, and appropriated (DeNora, 2017), without theoretical considerations of concepts as wellbeing and music, which in turn creates difficulties for both replication and evaluation.

From a salutogenetic perspective, experiences of coherence and meaning are perceived as prerequisites for an understanding of health and wellbeing (Antonovsky & Sagy, 2017), emphasizing opportunities for coping, physical and emotional agency, participation, and familiarity, along with the music interventions. Such practical and relational features are often visible in the collected results. Following this, findings display processes of health and wellbeing as working “through” the medium of music. This means musical activities underscore the vitalising of emotions, offering opportunities for coping, resilience and agency, participation, meaning and hope, especially important in childhood (Courtwright et al., 2020). Interpreted as such, shared musical activities are perceived as immunogens (Ruud, 2013). Depending on different theoretical frames, the conceptualization of music, health, and well-being, and their conceptual inter-relations, are not sufficiently outlined in all included studies. Nevertheless, concepts of health and wellbeing are subsequently used to present both objectives and findings, even if a few studies in this review lack enough transparency of design and methodology to be valid. When presenting interventions from music therapists, studies represented in this review often implicitly preclude the professional use of music as a medium for emotional regulation, coping and positive intersubjective experiences. Still, there is a subsequent lack of an explicit philosophical foundation for the medium of music in relation to health and wellbeing, and included studies thus show heterogeneous findings based on heterogeneous conceptualization. This further affects the reporting of both contexts and relations. In Preti and Welch (2011), one such implication is highlighted when stating that “the literature on the impact of music on hospitalized children does not offer integrated views that include a wider system of social and emotional interactions between the musicians, the child-patients, the caregivers, and the hospital staff” (p. 213–214).

## Design for participation

On a more practical level, subsequently referred to in the texts, social aspects seem an important ingredient of the live music interventions. At the core lies the accentuation of ´live´ interventions, compared to interventions with pre-recorded music, with the opportunity to monitor and adapt to the child’s present immediate needs (Bush et al., 2021; Giordano et al., 2020, 2021; Millett & Gooding, 2018; Scheufler et al., 2021; Văduva & Balla, 2019; Wong et al., 2021; Yates et al., 2018) giving space for intersubjectivity and participation from the child (Colwell et al., 2013; Scheufler et al., 2021; Uggla et al., 2016, 2018, 2019), supporting social skills and affecting family relations (Blackburn, 2020a, 2020b; Ortiz et al., 2019; Yates et al., 2018). In some of the studies, participative features are highlighted through the definition of social skills demanded from musicians while engaging child patients and their parents (C. Preti & G. F. Welch, 2013; C. Preti & G. Welch, 2013; Preti & Welch, 2011). In three of the included studies, the psycho-socio-cultural aspects of music intervention were not addressed. Here, reporting showed a view of music as having inherent qualities of comforting and altering the emotional state of the individual child patient (Antonelli et al., 2019; Grebosz-Haring & Thun-Hohenstein, 2018; Longhi et al., 2015). However, both Grebosz-Haring and Thun-Hohenstein (2018) and Longhi et al. (2015) reflected on the possibility of inherent social components of music interaction eventually affecting the result.

Although not entirely conceptually outlined in collected studies, participation still appears to be an important prerequisite of music intervention connected to health and wellbeing. Thus, following the core result, the adaptive structure of participation and play, through shared musical activities, offers space for inter-subjective, multimodal communication (non-verbal, physical, emotional), further supporting resilience, meaning, and coping strategies (Ruud, 2013). For example, following Blackburn (2020b), participative sensory-motor experiences may, depending on design, timeframe, and occurrence, even support the perception of ´normal life´, thereby reducing hospitalization, while at the same time strengthen limbs, body, and senses. Looking through these theoretical lenses, musical processes of health and wellbeing in paediatric hospital care seem favoured by a psycho-socio-cultural perception of the medium of music. In this way, live music intervention in paediatric hospital care shows itself to be an activity of importance both supporting and maintaining social connectedness throughout the hospital stay. Connected to the previously reported differences in outcomes between age groups, further reflections on the prerequisites for participation appear.

When discussing implications for design and performance, the question of age is rarely considered in the studies. Children 0–17 years are predominantly perceived as one group. Concerning outcomes of health and wellbeing from live music interventions, such a wide age range could easily be perceived as demanding when drawing conclusions for practice. A lack of reflection on age may also obstruct the opportunity to generalize, compare, and obtain outcomes among age-groups. Further, and coupled to a child- and family-centred perspective partly outlined in some of the studies (Blackburn, 2020a, 2020b; Millett & Gooding, 2018; Ortiz et al., 2019; Preti & Welch, 2011; Uggla et al., 2016, 2018, 2019; Yates et al., 2018), the findings circumscribing participation clearly promote relational aspects of music activities to be at the fore, occasionally using the definition of communicative musicality (Malloch et al., 2012) as a stepping stone for design and practice. With a more family-centred approach to treatment, music therapy may thus also be considered a vital resource to support quality of life for siblings and the whole family (Lindenfelser et al., 2012). Following this, a contextual understanding on music and health is at the fore. This means taking a perspective where musical artefacts and activities offer certain participative possibilities, in the moment. Perceived as such, “the health effects are not given but created through use by the involved participants of a situation” (Stige, 2013, p. 184).

## Emotional regulation

The core focus is on the impact of positive affect, measured through decreased stress, anxiety, and pain perception, displays the regulation of emotion through live music interventions as a prerequisite to reduce effects of hospitalization. Important to mention here is the use of both affect and emotion in the studies, where the concept affect is often generally used to explain measurable and viable effects on mood and energy, while the concept of emotion is used to explain the modification and regulation of feelings. Although some of the included studies did not discuss design, prerequisites or implications for positive affect, others reflected on at least one of these features. While attentively monitoring intervention, following the child’s mood and present state, this in turn is perceived to support coping, play, and participation. Important facilitators delineated here were adaptivity and familiarity giving the relational and emotional aspects of live music intervention great importance. This is also in line with a child- and family-centred perspective meaning that, especially for young children, family and social connectedness are vital for wellbeing, including overall positive emotions, positive self‐esteem, and resilience (Courtwright et al., 2020). Adolescents often have musical preferences of their own and usually search social connectedness even beyond family (Yousafzai, 2018), but a demanding hospital situation may nevertheless incidentally trigger the need for an increased emotional family support even for older children. Here participative art activities, such as live music interventions, may support experiences of belonging and wellbeing, also strongly emphasized in child rights (UNHCR) (Blackburn, 2020a). A child- and family-centred approach perceives family as a system, which in turn has implications for the design of the live music intervention (Davidson et al., 2017; Ullsten, 2019). For instance, supporting the mediating participation of parents may be an important step to establish a musical channel between musician and child (Blackburn, 2020b, p. 219). While focusing on emotional regulation for wellbeing through live music intervention, Uggla et al. (2018) argue that it is crucial to adapt to the child’s mood and state to keep the child’s affect-level within a window of tolerance, supporting the child to stay emotionally regulated. Again, with reference to age, for children younger than 18 months Uggla et al. (2018) emphasize the importance of working with the interaction between parents and the child, where intersubjective and affective communication needs to include the child´s body language. In summary, to work with health and wellbeing in paediatric hospital care by emotional regulation through participative musical activities is both in line with health perceived as a salutogenetic phenomenon, closely connected to physical, psychological, and social wellbeing (WHO) and music perceived as a social, cultural, and psychological medium (Elliott & Silverman, 2013; R. MacDonald et al., 2013). Following this, both conceptualizations have implications for health music practices where the very design of an adaptive intersubjective and participative intervention will influence opportunities for emotional regulation and further, features of health and wellbeing.

Approaching the question of profession, the category of emotional regulation displays a need for the training of certain professional skills (e.g., adaptivity, relational engagement, musical improvisation, the use of familiar repertoire) while working with music interventions with children and families in paediatric hospitals. However, the emotional and relational engagement demanded from musicians in charge poses questions concerning supervision and collegial reflections to manage the best outcomes possible in each situation (C. Preti & G. F. Welch, 2013; C. Preti & G. Welch, 2013). If live music interventions show themselves to be beneficial in supporting health and wellbeing in paediatric hospital care, these concerns may thus pose implications for the future organization of health music. Music therapists are professionally certified to work with music as a medium for health and wellbeing, while health musicians, still not perceived as a legitimate profession, may need more preparation for the use of the participative, relational, and emotional facets of the medium of music (Koivisto & Tähti, 2020), facets partly insufficiently outlined in the collected results in this review.

## Recommendations for practice

This scoping review, although synthetised findings show both great heterogeneity and recurrent methodological flaws, still shows some trends applicable for practice. According to our analysis, we highlight the need for:

- **A philosophical consideration** while implementing music for health and wellbeing in paediatric hospital care. This entails questioning the way stakeholders and professionals consider music as a medium for health. Is it aesthetic, social, psychological, communicative, physical, or participative? Also, which conceptualizations for health and wellbeing undermine this perception?
- **A focus on participation** which means considering the communicative features of live music activities for health through play, giving space for normalcy, children’s agency, resilience, and physical activation. This focus may also include a wider relational, child- and family-centred approach when using music interventions to create and maintain supportive structures and coping strategies.
- **A focus on emotional regulation** which means adaptive live musical interventions creating opportunities to follow and carefully monitor the child’s emotions through familiar and participative activities within their window of tolerance. To keep the child (and family) emotionally regulated may prevent PTSD and hospitalization.

## Strengths, limitations, and methodological considerations

As far as we are aware, there are no previous reviews of empirical research studying common outcomes of health and wellbeing connected to live music interventions, delivered by several professions, in paediatric in-patient hospital care settings. Due to the exploratory nature of a scoping review, both quantitative and qualitative studies may be qualitatively examined together, and the theoretical framework of this review showed itself to be helpful while interpreting the various study outcomes. At the same time, those theoretical lenses may certainly be perceived as having misrepresented certain findings, sometimes framing both objectives and results in a way not explicitly defined or reported in the studies. This may be seen as a lack of trustworthiness influencing both credibility of representation and transferability (Elo et al., 2014). Still, we argue that the transparency of objective, questions, and methodology of this review strengthen the quality of presented findings. Despite not being mandatory in scoping reviews, a quality appraisal was executed to meet previous scholarly concerns of methodological flaws in music interventions studies. As the studies included show great heterogeneity in design, methodology, and quality, it is difficult to draw clear conclusions. Nevertheless, through the qualitative content analysis, some important benefits for the practice of live music interventions in paediatric hospital care could be traced. One clear limitation is that studies found eligible for this study represent only publications in English with a high proportion from western European scholars and the US. Perspectives from non-Western European and non-American scholars are thus poorly represented except for one study from Rumania, one study from India and one from Singapore. Concerning geographical issues, the result shows both a variation in the quality of evidence, and certain areas where findings remain to be confirmed or better understood. One such issue is the lack of studies on health musicians, in the present review geographically represented solely by British and Italian scholars. Since collected findings mainly have their origin in occidental countries, the results still lend credibility that correspond to the triangulated result of the WHO’s European study of arts for health and wellbeing in which music and music therapy are treated on a more comprehensive level (Fancourt & Finn, 2019). Finally, the search was limited to peer-reviewed empirical articles, which may have excluded important contributions for practice from other publications (e.g., books, masters, and doctoral theses). When adopting a broad view of live music interventions in paediatric hospital care, we also chose to include subgroup perspectives. Due to the small number of studies, some perspectives are poorly represented and trustworthy conclusions on subgroup levels are thus difficult to draw.

## Conclusion

This scoping review began from an open and explorative point of departure while examining reported impacts and outcomes of live music interventions in paediatric hospital care published in empirical peer-reviewed studies during the past 10 years. The ambition was to chart findings, to illuminate needs for further research and to trace subsequent trends applicable to future practices. We found various conceptual flaws creating questions of a theoretical foundation circumscribing the combination of music and health, a philosophical theme currently increasing in contemporary scholarly conceptualization of music, health, and wellbeing. Despite heterogeneous design and methodology, partly due to epistemological matters, we still found some impacts more often represented than others. Positive affect (both quantitatively and qualitatively measured), coping and the possibility to reduce hospitalization showed themselves to be important features. Age and session design appeared when looking at beneficial aspects, barriers and facilitators, emotional regulation, questions of play and participation, adaptivity and familiarity. The communicative aspects of live music interventions in paediatric hospital care appear to be at the core of importance. Perceived in this way, findings have implications for future health practices with music, although heterogeneity of theory, design, and methodology accentuates the call for more rigorous design in future studies (Robb et al., 2011). To generalize and transfer findings to practice, a more carefully elaborated philosophical foundation for perceiving the medium of music as a health monitor may be of importance, both in future research studies and healthcare education. Nevertheless, despite the heterogeneity of design and quality, the collected findings indicate some promising keys for the future organization of live music interventions in paediatric hospital care. And, thanks to the different kinds of communicative opportunities displayed within and through the medium of music, “maybe this artform provides something relatively unique compared to other nonmedical activities? “ (Preti & Welch, 2011, p. 220).

## Notes

1. Encyclopædia Britannica. (n.d.). *Child development*. Britannica Academic. Retrieved March 30, 2022, from https://academic-eb-com.ezproxy.its.uu.se/levels/collegiate/article/child-development/24057.

2. Encyclopædia Britannica. (n.d.). *Adolescence*. Britannica Academic. Retrieved March 30, 2022, from https://academic-eb-com.ezproxy.its.uu.se/levels/collegiate/article/adolescence/3766.

3. WHO. Adolescent health. Retrieved March 30, 2022 from: https://www.who.int/southeastasia/health-topics/adolescent-health.
